# Prevention of allograft rejection in heart transplantation through concurrent gene silencing of TLR and Kinase signaling pathways

**DOI:** 10.1038/srep33869

**Published:** 2016-09-23

**Authors:** Hongmei Wang, Xusheng Zhang, Xiufen Zheng, Zhu Lan, Jun Shi, Jifu Jiang, Terry Zwiep, Qing Li, Douglas Quan, Zhu-Xu Zhang, Weiping Min

**Affiliations:** 1 Institute of Immunotherapy and Medical College of Nanchang University, and Jiangxi Academy of Medical Sciences, Nanchang, China; 2 Department of Surgery, Pathology, and Oncology, University of Western Ontario, London, Canada; 3 Multi-Organ Transplant Program, London Health Sciences Centre, London, Canada

## Abstract

Toll-like receptors (TLRs) act as initiators and conductors responsible for both innate and adaptive immune responses in organ transplantation. The mammalian target of rapamycin (mTOR) is one of the most critical signaling kinases that affects broad aspects of cellular functions including metabolism, growth, and survival. Recipients (BALB/c) were treated with MyD88, TRIF and mTOR siRNA vectors, 3 and 7 days prior to heart transplantation and 7, 14 and 21 days after transplantation. After siRNA treatment, recipients received a fully MHC-mismatched C57BL/6 heart. Treatment with mTOR siRNA significantly prolonged allograft survival in heart transplantation. Moreover, the combination of mTOR siRNA with MyD88 and TRIF siRNA further extended the allograft survival; Flow cytometric analysis showed an upregulation of FoxP3 expression in spleen lymphocytes and a concurrent downregulation of CD40, CD86 expression, upregulation of PD-L1 expression in splenic dendritic cells in MyD88, TRIF and mTOR treated mice. There is significantly upregulated T cell exhaustion in T cells isolated from tolerant recipients. This study is the first demonstration of preventing immune rejection of allogeneic heart grafts through concurrent gene silencing of TLR and kinase signaling pathways, highlighting the therapeutic potential of siRNA in clinical transplantation.

Heart transplantation has proven to be a successful therapeutic procedure for patients with end- stage heart failure. However, immunosuppressive drugs have to be used for the prevention of allograft rejection^1^. These drugs possess many potential adverse effects and also reduce the ability of recipients to fight against various infections and cancer, putting transplant patients at increased risk for both of these. Therefore, it is crucial to develop a new anti-rejection treatment that induces donor- specific tolerance without the need for continuous use of immunosuppressive drugs.

Toll-like Receptors (TLRs) are actively involved in graft rejection in transplantation activating the innate immune response and modulating adaptive immunity^2^. We have reported that blocking TLR signaling by targeting the TLRs adaptor molecules myeloid differentiation primary response gene (88) (MyD88) and TIR domain containing adaptor inducing IFNβ (TRIF) could prolong allograft survival. Furthermore, a combined use of rapamycin, the inhibitor of mammalian target of rapamycin (mTOR), further prolonged graft survival^3^.

mTOR is one of the most critical serine/threonine kinases that affects both innate and adaptive immunity. mTOR is composed of two distinct protein complexes, mTORC1 and mTORC2, and are respectively defined by the scaffold proteins regulatory-associated protein of mTOR (Raptor) and rapamycin-insensitive companion of mTOR (Rictor). While mTORC1 is proven to be rapamycin-sensitive, whether or not mTORC2 is directly inhibited by rapamycin is contestable. In the context of the immune system, stimuli from antigen receptors (T and B cell receptors), cytokine receptors (eg., IL-2 receptor) or TLRs lead to the activation of the PI3K-Akt-mTOR signaling pathway, which subsequently results in innate and adaptive immune responses. Accordingly, the suppression of mTOR using its specific inhibitor rapamycin prevents transplant graft rejection. Inhibition of mTOR suppresses the adaptive immunity via inhibiting and attenuating interferon gamma (IFN-γ) mediated Th1 as well as promoting regulatory T cells (Treg) generation^4^,^5^,^6^.

In addition to MyD88 and TRIF adaptor molecules, TLR agonists also activate the PI3K-Akt- mTOR pathway, another important downstream kinase responsible for modulation of TLR- induced proinflammatory and immune responses. Based on our previous study in which the combination of rapamycin and MyD88/TRIF siRNA significantly prolonged heart graft survival, we postulate that the inhibition of PI3K-Akt-mTOR pathway may enhance immune suppression; concurrently inhibiting mTOR may synergize MyD88/TRIF siRNA in tolerance induction in heart transplantation.

Specific silencing of genes using small interfering RNA (siRNA) is an advanced method of RNA interference that is more potent and specific in the knockdown of gene expression than conventional blocking methods^7^. Combined knocking down of TLRs adaptor molecules and mTOR may reduce the innate and adaptive immune response to the allograft and thus prolong allograft survival. In this study, we administrated MyD88, TIRF and mTOR siRNA expression vector to the recipient to examine whether this could significantly prolong cardiac allograft survival.

## Results

### mTOR, MyD88 and TRIF gene silencing *in vitro* in DCs

**Figure 1:**
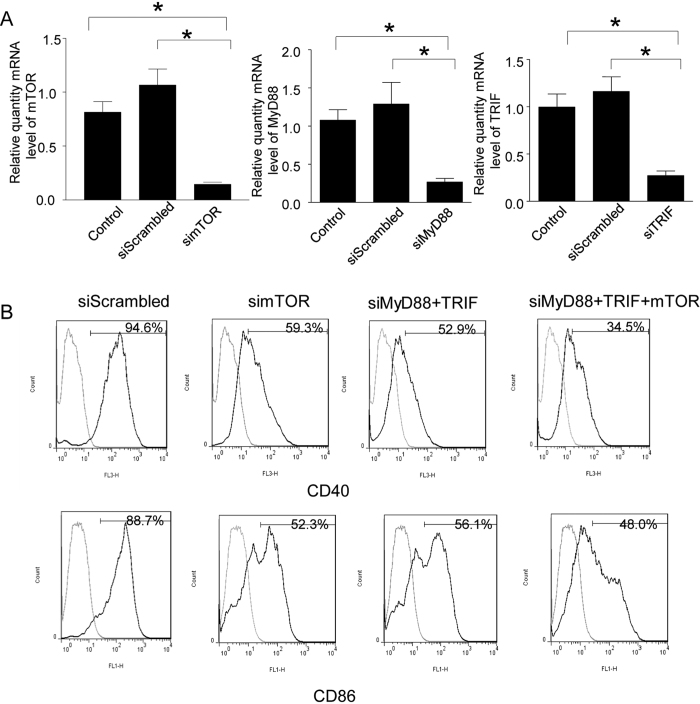
mTOR, MyD88 and TRIF gene silencing *in vitro*. (**A**) gene silencing *in vitro* determined by real time RT-PCR. C57BL/6 mice bone marrow DCs were cultured until day 6 and were transfected with mTOR, MyD88, TRIF siRNA or scrambled siRNA using lipofectamine 2000. Non-transfected cells used as negative control. Cells were harvested at 48 h post-transfection. Total RNA was extracted, relative quantity of mTOR, MyD88 and TRIF mRNA was detected by real time RT-PCR. (n = 4, *p < 0.01, mTOR, MyD88 or TRIF siRNA vs non- transfected or scrambled siRNA transfected cells.) (**B**) Concurrent knock down of TLR and mTOR pathway demonstrated a synergistic effect in reducing maturation of DCs. C57 BL/6 mice bone marrow DCs were cultured and transfected with MyD88, TRIF and mTOR siRNA using lipofectamine 2000. mTOR siRNA alone, MyD88 and TRIF siRNA and scrambled siRNA transfected cells serve as control. Twenty-four hours after transfection, LPS was added for another 24 h. DCs were harvested and stained FITC-labeled CD86, PE- Cy5 labeled CD40 antibodies. The expression of CD40 and CD86 was detected by flow cytometry. (n = 4 experiments for the data presented, p < 0.05, mTOR siRNA, MyD88 and TRIF siRNA or cocktail of 3 siRNAs vs scrambled siRNA transfected cells).

TLR and mTOR act as important regulators of the dendritic cells (DCs)’ maturation and function and play crucial roles in modulating both the innate and adaptive immune systems^6^,^8^,^9^. To confirm siRNA gene silencing efficacy, we transfected the cultured C57BL/6 mice bone marrow DCs with siRNA specifically targeting the mTOR, MyD88 and TRIF genes. Forty-eight hours after transfection, the expression of mTOR, MyD88 and TRIF genes was detected in the DCs by quantitative real time RT-PCR (Fig. 1A). mTOR, MyD88 and TRIF genes expression was significantly knocked down by 75–80% when compared with the DCs transfected with scrambled siRNA, or untransfected negative control DCs. (Fig. 1A).Therefore, we confirmed the gene silencing efficacy of siRNAs specifically targeting the mTOR, MyD88 and TRIF genes.

### Concurrent silencing of TLR and mTOR pathway has a synergistic effect in the reduction of DC maturation and increased negative regulator PD-L1 expression

TLRs on DCs identify specific structures of microorganisms (pathogen-associated molecular patterns PAMPs), recruit intracellular adaptors, MyD88 and TIRF, and lead to DC maturation. We demonstrated that silencing both MyD88 and TRIF genes resulted in reducing DC maturation^3^. It had been reported that rapamycin, an mTORC1 inhibitor, reduces DCs co- stimulatory molecules expression and impairs their function^10^. We therefore explored whether concurrent silencing of both TLR and mTOR signaling pathways has a synergistic effect in the reduction of DCs maturation. DCs were cultured from bone marrow progenitor cells, and then transfected with mTOR siRNA alone, MyD88 and TRIF siRNA or mixture of mTOR, MyD88 and TRIF siRNA. DCs were transfected with scrambled siRNA as a control. Twenty-four hours after transfection, the transfected DCs were stimulated with LPS overnight. We tested the costimulatory molecules, CD40 and CD86, expression of the DC by flow cytometry in different treatment groups. Control DCs that were transfected with scrambled siRNA highly expressed CD40 (94.6%) and CD86 (88.7%), suggesting that these DCs were mature (Fig. 1B). Compared to control DCs, transfection with MyD88 and TRIF siRNA or mTOR siRNA alone both can reduce CD40 (52.9%, 59.3% vs 94.6%) and CD86 (56.1%, 52.3% vs 88.7%) expression. Concurrent silencing of MyD88, TRIF and mTOR genes had a synergistic effect leading to further reduction of CD40 (34.5%) and CD86 (48.0%) expression (Fig. 1B).

**Figure 2:**
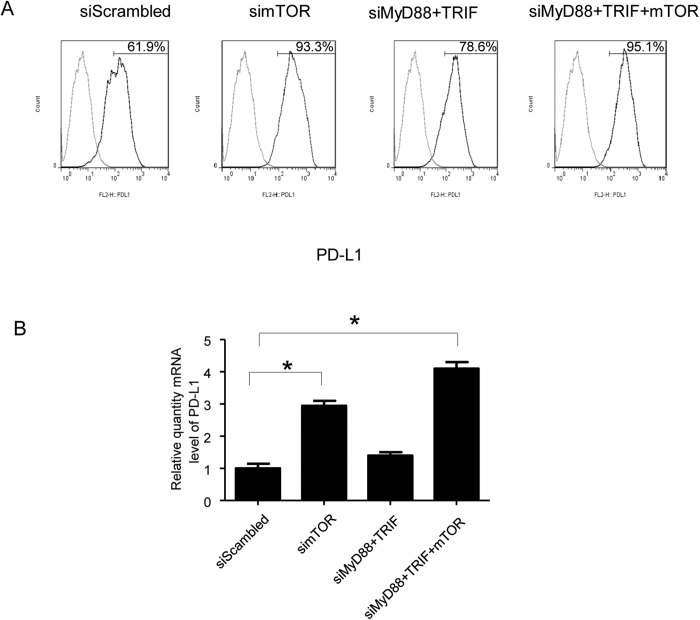
Silencing mTOR gene in DCs increased PD-L1 expression. (**A**) Bone marrow DCs were cultured and transfected with MyD88, TRIF and mTOR siRNA as described in Fig. 1. Forty-eight hours after transfection, DCs were collected and stained with PE-labeled PD-L1 antibody. The expression of PD-L1 was detected by flow cytometry. (n = 4 experiments for the data presented, p < 0.05, mTOR siRNA or combined with MyD88 and TRIF siRNA vs scrambled siRNA transfected cells). (**B**) The total RNA of DC was extracted and relative quantity of PD-L1 mRNA was detected by Real-Time RT-PCR. (n = 4 experiments, *p < 0.05, mTOR siRNA or combined with MyD88 and TRIF siRNA vs scrambled siRNA transfected cells).

DCs perform as professional antigen presenting cells and provide positive or negative signals to regulate T cells function. Programmed death ligand 1 (PD-L1) expressed on DCs binds with Programmed cell death protein 1 (PD-1) on T cells and negatively regulates T cells activity and results in a lack of T cell response to the antigen^11^. Rosborough *et al*., reported that Torin1 conditioned DCs which block both mTORC1 and mTORC2 expressed elevated levels of PD-L1^12^. We also found that silencing mTOR in DCs significantly increased PD-L1 expression at the protein level by flow cytometry compared with control siRNA and MyD88/TRIF silenced DCs (93.3% vs 61.9% and 78.6%). Combination of MyD88, TRIF and mTOR siRNA silencing presented an additive effect on PD-L1 expression in DCs andPD-L1 expression in tripled silenced group was 95.1% (Fig. 2A). The results were also confirmed by real time RT-PCR in which PD-L1 expression increased 2.9 and 4.1 folds at the mRNA level in mTOR siNRA alone or combined with MyD88/TRIF siRNA silenced DCs compared with scrambled siRNA treated DCs (Fig. 2B).

These data show that concurrent silencing of both TLR and mTOR signaling pathways has a synergistic effect in reducing DCs maturation and increasing negative regulator PD-L1 expression.

### TLR and mTOR silenced DCs suppress allogeneic T cell proliferation and induce Treg generation

**Figure 3:**
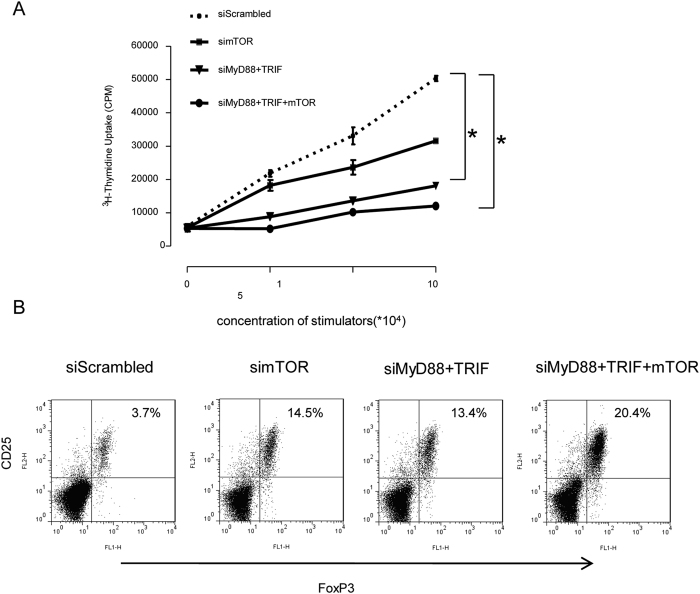
TLR and mTOR silenced DCs further suppress allogeneic T cell proliferation and promote more Treg cell generation. (**A**) Bone marrow-derived DCs were cultured and transfected with MyD88, TRIF and mTOR siRNA as described in Fig. 1. Forty-eight hours after transfection, DCs were collected and co-cultured with allogeneic T cells in a 96 well plate at various ratios as indicated. [3H]-thymidine was added 48 h after co-culture, and the following day, its incorporation was measured as an indicator of T cell proliferation. (n = 4 experiments, *p < 0.01 vs control group). (**B**) Forty-eight hours after transfection, DCs were collected and co-cultured with allogeneic T cells in a 24 well plate for 7 days. The cells were collected and stained with FITC-labeled FoxP3, PE-labeled CD25, PE-Cy5 labeled CD4 antibodies. Flow cytometry was performed to demonstrate percentage of CD25^+^FoxP3^+^ expression in CD4+ allogeneic T cells. (n = 4 experiments for the data presented, p < 0.01, mTOR siRNA, MyD88 and TRIF siRNA or cocktail of 3 siRNAs vs scrambled siRNA transfected cells).

We next sought to determine the function of DCs after gene silencing of TLR and mTOR signaling pathways using mixed lymphocyte reaction (MLR) to test allogeneic T cells stimulatory ability of siRNA-treated DCs. DCs cultured from C57BL/6 mice were transfected with MyD88 and TRIF siRNA, and mTOR siRNA alone or in combination and were used as stimulators. DCs transfected with scrambled siRNA was used as controls. These DCs were cultured with allogeneic T cells from BALB/c mice. The results demonstrated that, compared with scrambled siRNA-transfected DCs, mTOR siRNA alone silenced DCs reduced levels of allogeneic T cell proliferation. Silencing both MyD88 and TRIF using siRNA significantly inhibited allogeneic T cell proliferation. Combined silencing of TLR and mTOR pathways showed a synergistic effect in restraining allogeneic T cells proliferation (Fig. 3A).

We further explored the ability of siRNA silenced DCs to induce Treg. Compared with scrambled siRNA transfected DCs, mTOR siRNA alone and MyD88 plus TRIF siRNA transfected DCs increased Treg induction. The percentage of FoxP3+CD25+ population in CD4+ cells was 14.5% and 13.4% respectively, while in allogeneic T cells cocultured with scrambled siRNA transfected DCs, the percentage of FoxP3+ CD25+ was only 3.7%. Concurrent silencing of MyD88, TRIF and mTOR genes in DCs had a synergistic effect on the induction of Treg as 20.4% of CD4+ cells were FoxP3+CD25+ (Fig. 3B). The results suggested that silencing both TLR and mTOR signaling pathways in DCs significantly reduced their ability to stimulate allogeneic T cells and induced more Treg generation.

### mTOR and TLR adaptors silenced DCs induced allogeneic T cells exhaustion

**Figure 4:**
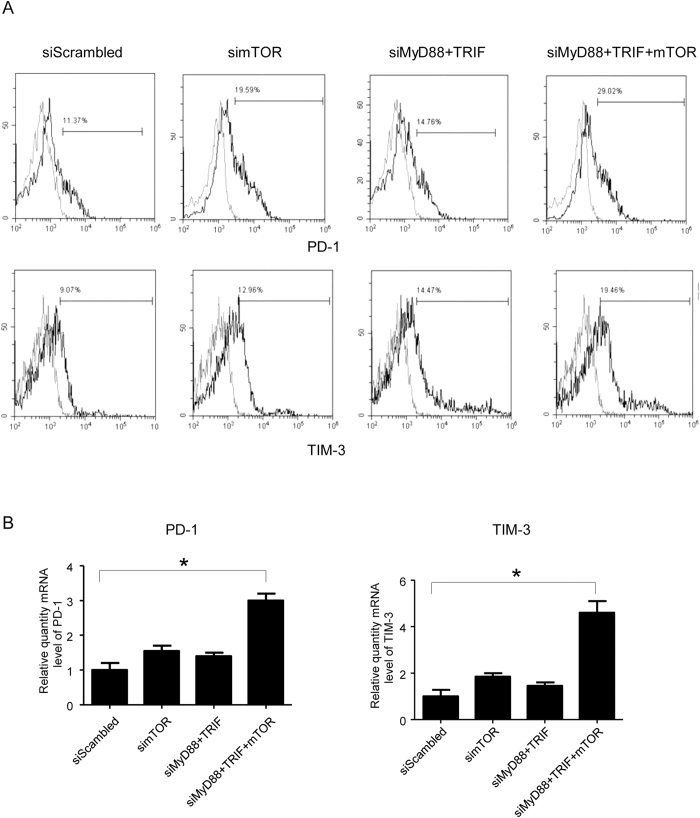
TLR and mTOR silenced DCs induced allogeneic T cell exhaustion. Bone marrow-derived DCs were cultured and transfected with MyD88, TRIF and mTOR siRNA as described in Fig. 1. Forty-eight hours after transfection, DCs were collected and co-cultured with allogeneic T cells in a 24 well plate for 5 days. The cells were collected and stained with Percp-efluro710 labeled PD-1 and PE-labeled TIM-3 antibodies. Flow cytometry was performed to determine PD-1 and TIM-3 expression in T cells (A). The total RNA of co-cultured cells was extracted, relative quantity of PD-1 and TIM-3 mRNA was detected by real time RT-PCR. (n = 4, *p < 0.01, mTOR, MyD88 and TRIF siRNA vs scrambled siRNA transfected DCs).

Co-stimulatory and co-inhibitory receptors play key roles in T cell activation or dysfunction^13^,^14^. T-cell exhaustion is a state of T-cell dysfunction and exhausted T cells lose robust immune response functions^15^,^16^. PD-1, one of the T cell exhaustion markers and its ligands, PD-L1 and PD-L2 is one of the critical inhibitory pathways for inducing allograft tolerance in the murine transplantation model^17^. As our results demonstrated that silencing DCs with mTOR siRNA significantly increased PD-L1 expression in DCs, we further explored whether mTOR siRNA treated DCs will induce allogenenic T cell exhaustion or not. DCs cultured from C57BL/6 mice were transfected with MyD88 siRNA and TRIF siRNA, mTOR siRNA alone or in combination and were cocultured with allogenic T cells from BALB/c mice for 5 days. DCs treated with scrambled siRNA were used as controls. The cells were collected and PD-1 expression was detected by flow cytometry. The results demonstrated that compared with scrambled siRNA transfected DCs, mTOR or MyD88 plus TRIF siRNA transfected DCs increased PD-1 expression in cocultured allogenic T cells (19.6%, 14.8% vs 11.4%). The combination of siRNA treated DCs further increased PD-1 expression to 29.0% (Fig. 4A).

T cell immunoglobulin and mucin domain-containing protein 3 (Tim-3) is also an inhibitory receptor which is expressed on the surface of exhausted T cells. A previous study showed that CD80/CD86 ^lo^ DCs promoted expression of both PD-1 and TIM-3^18^. Our results demonstrated that DCs treated with mTOR, MyD88 plus TRIF siRNA increased TIM-3 expression in allogeneic T cells after 5 days of coculture, as compared with DCs transfected with scrambled siRNA. Allogenic T cells TIM-3 expression increased to 19.5% when cocultured with DCs treated with combination siRNAs (Fig. 4A).

The results were also confirmed by real time RT-PCR. The mRNA expression of PD-1 and TIM-3 in allogeneic T cells cocultured with combined siRNA transfected DCs increased 3.0 and 4.5 fold respectively, compared with T cells cocultured with scrambled siRNA transfected DCs. (Fig. 4B). Taken together, these results indicated that mTOR and TLR adaptor silenced DCs can induce allogeneic T cell exhaustion and may promote tolerance in transplantation.

### Prevention of cardiac allograft rejection by silencing both TLR adaptors and mTOR genes with siRNA expressed vector

**Figure 5:**
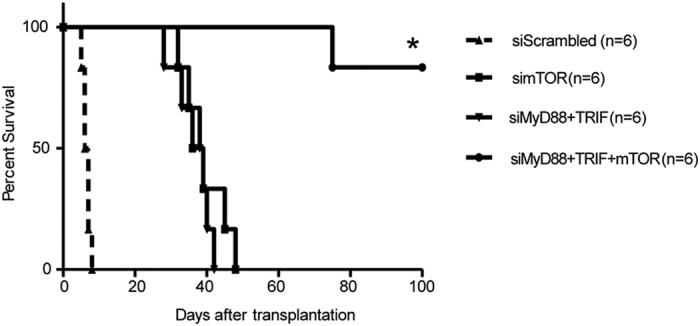
Prevention of allograft rejection through knocking down the TLR and mTOR pathway. Recipient BALB/c mice were injected with 50 μg MyD88, TRIF and mTOR expressed siRNA vectors by hydrodynamic injection through the tail veins. MHC fully mismatched allogeneic cardiac transplantation was performed from C57BL/6 mice to BALB/c mice. At 7, 14 and 21 days after transplantation, mice were treated with 50 μg MyD88, TRIF and mTOR siRNA vectors. The groups of mice that treated with MyD88 and TRIF siRNA vector, mTOR siRNA alone, or scrambled siRNA were used as controls (n = 6, *p < 0.05 vs control groups).

We reported that interruption of the TLR signaling pathway with low dose rapamycin can increase allograft survival^3^. Rapamycin is a potent mTORC1 inhibitor and acts as an immunosuppressant and anti-cancer agent^19^. *In vitro* results show that concurrent gene silencing of TLR and mTOR genes has a synergistic effect in reducing DC maturation and increased PD-L1 expression (Figs 1B and 2A,B) and inhibits allogeneic T cell proliferation promoting more Treg generation (Fig. 3A,B) T cells exhaustion (Fig. 4A,B). We, therefore, hypothesized that blocking both the TLR and mTOR signaling pathways might induce long term allograft survival. To test this, we treated BALB/C recipients with MyD88, TRIF and mTOR expressed siRNA vectors before fully MHC- mismatched transplantation of C57BL/6 hearts was performed. In the control group of recipients treated with scrambled siRNA, the allograft only survived 5–8 days. Treatment with either MyD88 and TRIF siRNA, or mTOR siRNA alone, significantly prolonged cardiac allograft survival (36.7 ± 2.1 days and 39.2 ± 2.5 days) (Fig. 5). Furthermore, combined silencing of MyD88, TRIF and mTOR genes further increased allograft survival (95.8 ± 4.6 days); 85.7% of recipients achieved acceptance of allografts (Fig. 5).

### Knockdown of TLR adaptors modulators and the mTOR signaling pathway induce more Treg generation *in vivo*

**Figure 6:**
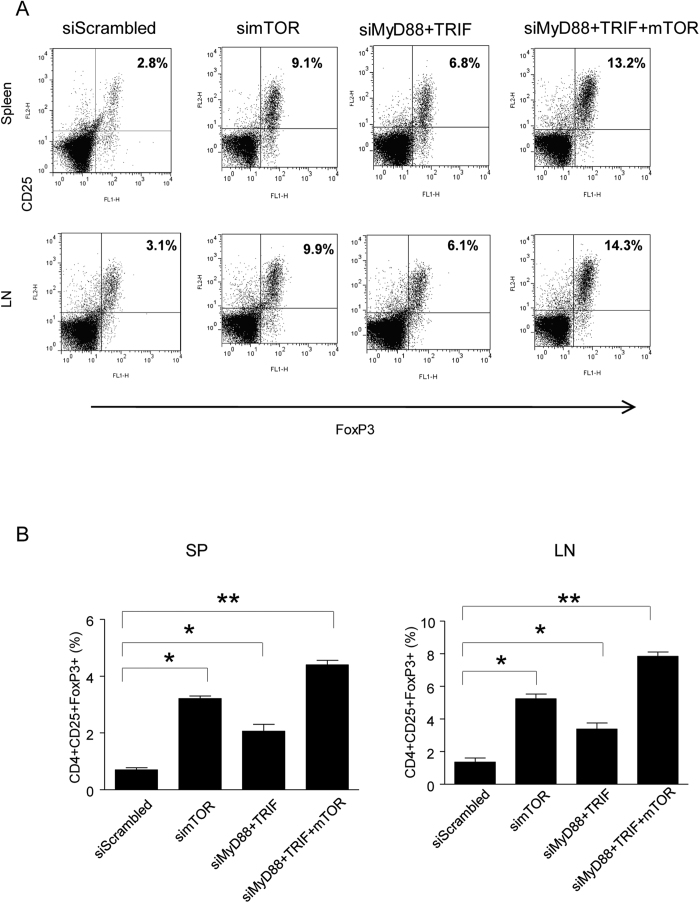
Treg cells in cardiac allograft recipients. (**A**,**B**) Flow cytometric analysis of Treg cells in BALB/C recipients of C57BL/6 hearts. T cells were isolated from spleens and lymph nodes of recipient mice 7 days post transplantation. T cells were stained with monoclonal antibodies against FoxP3, CD25 and CD4. Flow cytometry was performed to determine the percentages of Treg cells by first gating CD4+ cells and then subsequently analyzing the percentages of CD25+ FoxP3+ cells in spleen and LN from the recipient mice (**A**), and the average percentage of CD4+CD25+FoxP3+ Treg cells population (**B**) (n = 6, **P < *0.01*, *P < *0.05* vs the control group).

Treg plays a critical role in inducing and maintaining tolerance in organ transplantation^20^. TLRs are expressed on DCs and T cells and they can modulate Treg generation through direct action or indirectly through DCs^21^,^22^,^23^. It has been reported that inhibition of mTOR promotes Treg generation^24^,^25^,^26^. We presumed that treatment of the recipient with MyD88, TIRF and mTOR siRNA expression vectors in order to prolong allograft survival may accompany more Treg cell generation. To test that, we detected Treg in the spleen and Lymph node (LN) of the recipient with different treatments. The results demonstrated that compared to the recipient treated with scrambled siRNA, recipients treated with mTOR siRNA alone or MyD88 plus TRIF siRNA can increase the percentage of CD4+CD25+FoxP3+ T cells in both the spleen and LN (Fig. 6A,B). Concurrent silencing of both TLR adaptors and mTOR pathway had a synergistic effect of inducing significant Treg generation and long term allograft survival (Fig. 6A,B).

### DCs in tolerant recipients are immature and suppress allogeneic T cell proliferation

**Figure 7:**
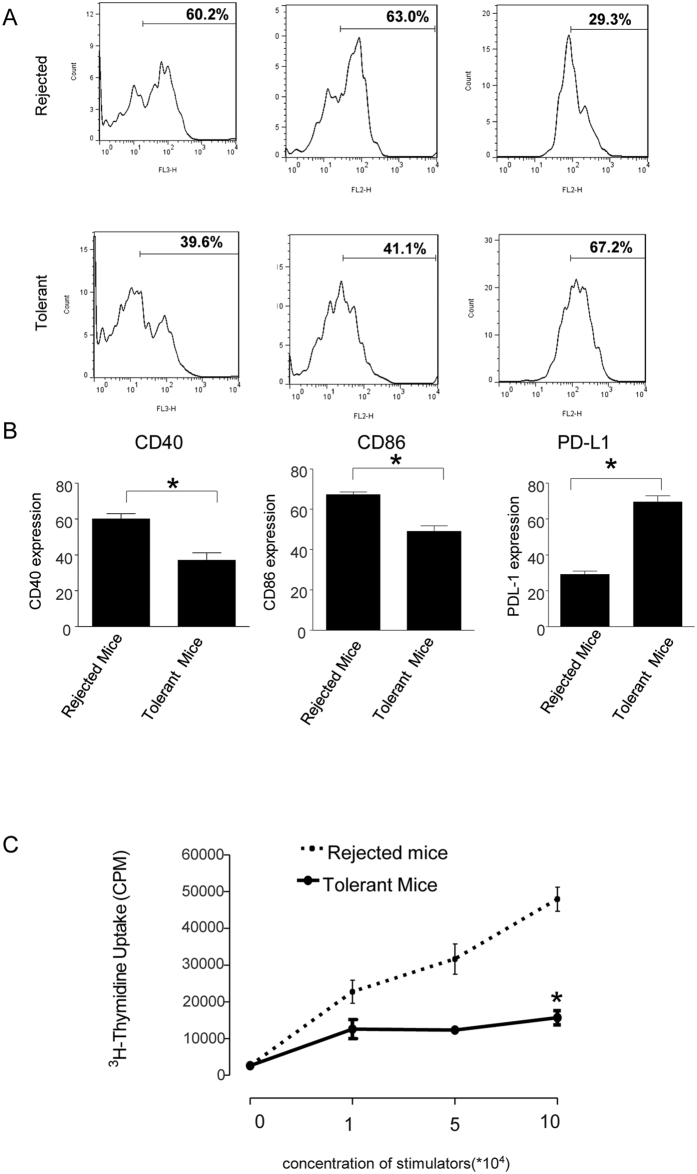
Immune modulations in tolerant recipients after MyD88, TRIF and mTOR siRNA treatment. (**A**,**B**) Tol-DC generated in tolerant recipients. Mice were treated and transplanted with allografts as described in Fig. 5. Splenic cells were isolated from BALB/c recipients at the time of rejection or 100 days post transplantation. Splenic cells were stained with antibodies against CD11C, CD40, CD86 and PD-L1. Flow cytometry was performed to determine the splenic DCs population by first gating CD11C+ cells and then subsequently analyzing the CD40, CD86 and PD-L1 expression of recipient mice (A), and the average expression of CD40, CD86 and PD-L1 (**B**) (n = 6, *P < *0.05* vs control group). (**C**) DCs from long-term survival recipients attenuate alloimmune stimulatory capacity. Mice were treated and transplanted with allografts as described in Fig. 3. Splenic DCs were isolated from BALB/c recipients at the time of rejection or 100 days post transplantation. DCs were co-cultured with allogeneic T cells from naïve C57BL/6 mice at different ratios. After 48 h, [3H]-thymidine was added to the coculture for another 18 h, and its incorporation was measured as an indicator of T cell proliferation. Data shown are mean ± SEM (n = 6, *P < *0.05* vs control group).

In transplantation, DCs play a key role in directing the alloimmune response and depend on the state of DCs to direct allograft tolerance or rejection^27^. To determine the state of DCs in recipients, CD40, CD86, and PD-L1 expression was examined. In rejected recipients, there was a high expression of CD40 (60.2%) and CD86 (63.0%), however in tolerant recipients, CD40 (39.6%) and CD86 (41.1%) expression were significantly decreased. On the contrary, tolerant recipients had a higher level of PD-L1 expression compared to rejected recipients (67.2% vs 29.3%, Fig. 7A,B). DCs with low CD40, CD86 and high PD-L1 expression induced antigen specific Treg generation. We next tested the function of the splenic DCs. The splenic DCs from recipients with rejected allografts displayed a vigorous stimulation of allogeneic T cell proliferation. In contrast, in tolerant recipients with silencing of both TLR adaptors and the mTOR signaling pathway, splenic DCs had significantly inhibited allogeneic T cell proliferation in a MLR (Fig. 7C). These data suggest that concurrent silencing of both TLR adaptor and mTOR signaling pathway generate more powerful tolergenic DCs as they suppress the allogeneic T cell response and may provide the conditions to generate more antigen specific Treg and induce immune tolerance.

### T cell exhaustion was increased in tolerant recipients

**Figure 8:**
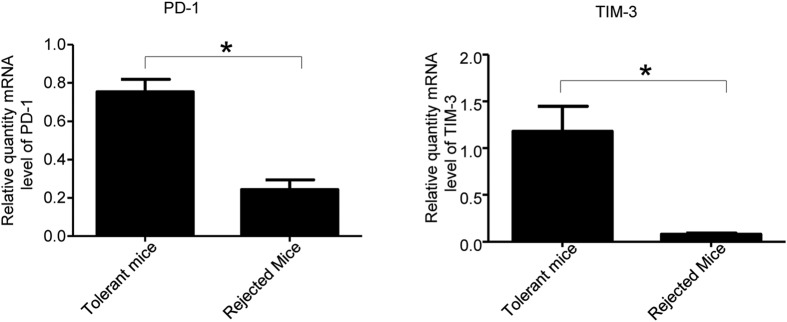
T cell exhaustion in tolerant recipients after MyD88, TRIF and mTOR siRNA treatment. Mice were treated and transplanted with allografts as described in Fig. 5. The splenic T cells were isolated from different experimental groups as indicated. RNA was extracted, and transcripts of PD-1, TIM-3 were determined using real time RT-PCR. Data shown are mean ± SEM (n = 6, *P<0.01 vs control groups).

We detected T exhaustion markers PD-1 and TIM-3 in the tolerant and rejected recipients. At the endpoint of experiment, the splenic T cells were isolated from recipients. Levels of PD-1 and TIM-3 were detected by quantitative real time RT-PCR. Tolerant mice treated with both TLR adaptors and mTOR siRNA showed elevated levels of PD-1 and TIM-3 gene expression compared with rejected recipients (Fig. 8).

## Discussion

Induction of immune tolerance that results in permanent acceptance of allogeneic grafts without immune rejection is a lofty goal for transplantation. Many attempts have been done to generate transplant tolerance, and limited success has been achieved in animal models. In the past decade, we have developed multiple regimens of transplant tolerance to prevent graft rejection through immune modulation and gene silencing. There are a series of immune modulatory events that can induce different states of T cell dysfunction including tolerance, exhaustion, anergy, senescence, deletion and ignorance leading to transplant tolerance^28^. These different types of T cell dysfunction can occur simultaneously in transplant tolerance^15^. On the other hand, activation of naive T cells is highly dependent on three signals between antigen presenting cells (APC) and T cells, including antigenic stimulation through the T cell receptor (TCR) and major histocompatibility complex II (MHC II) on the APC, costimulatory molecules and inflammatory cytokines. Positive costimulatory pathways including CD28:B7, CD40:CD154, OX40:OX40L promote complete T-cell activation and development of effector function^29^. Blocking the positive costimulatory pathways, TCR signaling alone resulted in T cell dysfunction and prolonged allograft survival in transplantation^30^. By contrast, negative costimulatory pathways, including cytotoxic T-lymphocyte-associated protein 4: B7 (CTLA-4:B7), PD-1:PD-L1/PD-L2, Herpesvirus entry mediator: B- and T-lymphocyte attenuator (HVEM:BTLA/CD160), and TIM-3:Galectin-9, downregulated T cell proliferation and induced antigen specific tolerance^31^.

T cell exhaustion was initially described as dysfunction of T cells during chronic infections and cancer. Induction of T cell exhaustion is a just recognized emerging mechanism of transplant tolerance which may contribute significantly to transplant survival^15^,^28^,^32^. Both extrinsic negative regulatory pathways (such as immunoregulatory cytokines) and cell-intrinsic negative regulatory pathways (such as PD-1) play key roles in T cell exhaustion^33^. Exhausted T cells are characterized by expression of several transcription factors and inhibitory receptors (iRs), such as PD1, TIM3, BTLA, CTLA-4, Lymphocyte-activation gene 3 (LAG3), 2B4, CD160, and Killer cell lectin-like receptor subfamily G member 1 (KLRG1), contributing to their poor functional state^34^,^35^,^36^,^37^,^38^. In this study, we demonstrated that gene silencing of TLR adaptors (MyD88/TRIF) and mTOR can delay immune rejection in heart transplantation, which is associated with T cell exhaustion (Fig. 8), suggesting that T cell exhaustion may at least partially attribute to immune tolerance and that synergized immune modulation occurs through the interaction of TLR adaptors and kinase signaling pathways.

The prolonged and/or high expression of multiple upregulated iRs in T exhausted cells play an important role in autoimmunity and transplant tolerance^39^,^40^,^41^,^42^. PD-1 and PD-L1/PD-L2 pathway is a major and best studied iRs pathway involved in T cell exhaustion. PD1 is a type I transmembrane receptor and is member of the immunoglobin gene superfamily. It is also a member of the CTLA-4 family of T-cell regulators and is expressed on the surface of T cells, B cells, macrophages, and DCs. PD-1 interacts with two ligands, PD-L1/PD-L2 which are expressed on APC and other immune cells. PD-1 and PD-1L interaction inhibits T-cell activation and cytokine production. PD-L1 is a transmembrane protein and has been presumed to play a critical role in transplant tolerance and autoimmune disease. In murine transplantation models, administration of anti-PD-L1 antibodies or lacking of PD-L1 on donor tissue accelerated allograft rejection or abrogated tolerance induced by CTLA4Ig^43^. Rosborough *et al*. reported that by contrast with rapamycin, inhibition of both mTORC1 and mTORC2 in DCs elevated PD-L1 expression^12^. In agreement with this notion, we used mTOR siRNA to transfect DCs and found PD-L1 expression in mTOR silenced DCs was increased at protein and mRNA levels (Fig. 2A,B). A recent study showed that increasing donor antigen specific T cells exhaustion may provide a novel strategy to prolong allograft survival and induce transplantation tolerance^44^.

Tim-3 is also expressed on exhausted T cells, and may regulate alloimmune responses and significantly prolong allograft survival in heart and skin transplantation through Tim-3: Galectin-9 inhibitory pathway^45^,^46^. Bauer *et al*., reported that activation of cytotoxic T lymphocytes (CTLs) by Treg-conditioned CD80/86^lo^ DCs increased expression of both TIM-3 and PD-1^18^. In our study, we knocked down TLR adaptor and mTOR gene expression in DCs and observed lower CD40 and CD86 expression, and promoted T cells increased both PD-1 and Tim-3 expression *in vitro* (Fig. 4A,B). In tolerant recipients, we also found that both PD-1 and Tim-3 expression was increased compared with rejected recipients. The results further confirmed that T cell exhaustion may be an important part of the mechanism for transplant tolerance and also implied the involvement of iR ligands in immune tolerance induced by the blockage of mTOR in heart transplantation.

In our previous study, we demonstrated that silencing of MyD88 and TRIF genes impairs DC maturation, inhibits allogeneic T cell proliferation, and promotes Treg generation, and that combined treatment with rapamycin induced allograft survival in heart transplantation^3^. Rapamycin is a specific inhibitor of mTORC1. Studies have reported that mTORC1 is important for Th1 and Th17 differentiation and mTORC2 is critical for Th2 differentiation^47^,^48^,^49^. Blocking both of mTORC1 and mTOC2, not only inhibited differentiation of Th1, Th2 and Th17 but also promoted more FoxP3+ Treg generation^12^,^50^,^51^. Compared with rapamycin that selectively inhibits mTORC1, mTOR inhibition suppressed the effector T cell activation and promoted Treg generation, but did not affect the function and homeostasis of Treg^6^. mTOR inhibition suppresses IL-4 dependent mouse bone marrow DCs maturation and inhibition of both signaling decreases positive costimulatory molecules expression in DCs^52^. It has been reported that a new generation of mTOR kinase inhibitor which blocks both of mTORC1 and mTORC2 had more potent immunosuppressive function and prolonged allograft survival in rodent organ transplantation models^51^,^53^,^54^. In our study, we used siRNA targeting the mTOR gene to knock down mTOR which is a component for both mTORC1 and mTOCR2, subsequently not only inhibited mTORC1, but also blocked mTOR2. From this aspect, mTOR siRNA seems more powerful than rapamycin for inducing tolerance in transplantation. We found that the silencing mTOR gene in DCs decreased CD40 and CD86 expression in DCs (Fig. 1B) and increased PD-L1 (Fig. 2A,B), and that combined knocking down of TLR adaptor genes and mTOR had a synergetic effect on decreasing expression of positive costimulatory molecules. Moreover, our results showed that there was more Treg generation in the recipient mice treated with mTOR siRNA vector, as compared with scrambled siRNA vector treated mice (Fig. 6A).

Many studies revealed that long term use of mTOR inhibitors including rapamycin produces lots of side effects such as oral mucositis, stomatitis, diarrhea, noninfectious pneumonitis, diabetes, nephrotoxicity, delayed graft function and gonadal toxicity^55^,^56^,^57^,^58^,^59^,^60^. Compared with immunosuppressive drugs, siRNA has been reported with lower toxicity, which makes it suitable for clinical therapy^61^. Furthermore, our previous study has demonstrated there is an inhibitory feedback loop between tolerogenic DCs and Treg cells *in vitro* and *in vivo*^62^. In this study, we administrated siRNAs in 3 weeks after transplantation to knock down mTOR and TLR adaptor genes resulting in induction of tolerogenic DCs and Tregs. The generated tolerogenic DCs and Tregs formed self-maintaining inhibitory loop and induced donor-specific immune tolerance, which waives the long term use of immunosuppression and minimize the side effects of systemic immune inhibition. Nevertheless, future study on potential toxicity of siRNA is needed in order to translate this research finding in to clinic.

In conclusion, our study demonstrates that silencing of TLR adaptor and mTOR genes impairs DC maturation and promotes Treg cell generation, and increases PD-L1 expression and T cell exhaustion, thereby preventing immune rejection in heart transplantation. These results highlight the therapeutic potential of siRNA in clinical transplantation.

## Methods and Material

### Mice and heterotopic cardiac transplantation

Male C57BL/6 (H-2b) and BALB/c (H-2d) mice (Charles River Canada, Saint-Constant, Canada) weighing 25 to 30 g were used as donors and recipients, respectively. All experiments in the study were performed in accordance with the guidelines established by the Canadian Council of Animal Care and were approved by the Animal Care Committee of the University of Western Ontario.

Recipients (BALB/c) were treated with MyD88, TRIF and mTOR siRNA expression vectors 7 and 3 days prior to heart transplantation and 7, 14 and 21 days after transplantation by hydrodynamic injection. Fifty micrograms of each MyD88, TRIF and mTOR siRNA vectors were diluted in 1.6 ml of PBS and rapidly injected into the mice through the tail vein within 5–7s. Recipient BALB/c mice were subjected to intra-abdominal allogeneic cardiac transplantation using the hearts from fully MHC-mismatched C57BL/6 mice according to our laboratory’s routine procedure. Pulsation of cardiac grafts was monitored daily by direct abdominal palpation in a double-blind manner to determine survival/rejection of the cardiac graft.

### DCs culture and transfection

DCs were cultured from bone marrow progenitor cells as previously described^63^. Briefly, bone marrow cells were flushed from the femurs and tibias of C57BL/6 mice, then washed and cultured in 6-well plates supplemented with 10 ng/ml of recombinant Granulocyte-macrophage colony-stimulating factor (GM-CSF) and recombinant mouse IL-4 (Peprotech, Rocky Hill, NJ, USA). All cultures were incubated at 37 °C in 5% humidified CO_2_. Non-adherent cells were removed (Day 2) and fresh medium was added. Medium were changed every 2 days, until day 6 for transfection. The DCs were transfected with siRNA by using lipofectamine 2000 (Life technologies, Burlington). Twenty-four hours after transfection, Lipopolysaccharide (LPS, 100 ng/ml) was added to the medium overnight and then the cells were recollected for further experiments.

### MyD88, TRIF and mTOR siRNA and expressed siRNA vector constructs

For *in vitro* studies, MyD88, TRIF and mTOR siRNA were synthesized by Dharmacon (Ottawa, ON). The sequences of MyD88 and TIRF siRNA are as previous described^3^. mTOR siRNA was purchased from Cell Signaling Technology (Whitby, ON, cat#6332S).

For *in vivo* studies, the siRNA expression vector was constructed as previously described^64^,^65^. The oligonucleotides containing target-specific sense and anti-sense sequences of MyD88, TRIF and mTOR mRNAs were synthesized, annealed and inserted into the pRNAT H1.1 siRNA expression vector utilizing restriction enzyme sites at the end of the strands (Genscript, Piscataway, NJ) to express the siRNAs.

### Quantitative real time RT-PCR

Total RNA was extracted from cells using Trizol (Invitrogen). RNA was reverse-transcribed using oligo-(dT) primer and reverse transcriptase (Invitrogen). Primers used for the amplification of murine mTOR, MyD88, TIRF, PD-1, TIM-3, PD-L1 and GAPDH genes were as follows: mTOR, 5′-CCACACAGCGTGATTGACTAT-3′ (forward) and 5′-GCAGCC GATGAGAGACAGAT-3′ (reverse); MyD88, 5′-TAGACCGTGAGGATATACTGAAGG-3′ (forward) and 5′-TTAGCTCGCTGGCAATGG-3′ (reverse); TRIF, 5′-ATGGGCCCAGCAAGCTATGTAAC-3′ (forward), 5′-TAGGGGAGGCTTGGAGGGATGGT-3′ (reverse); PD-1, 5′-CCGCTTCCAGATCATACAG-3′ (forward), 5′-CTCTGGCCTCTGACATACTTG-3′ (reverse); TIM3, 5′-CGGAGGTCGGTCAGAATGCCTATC-3′ (forward), 5′-GGGCT CCTCCACTTCATATACGTTC-3′ (reverse), PD-L1, 5′-GACCAGCTTTTGAAGGGAAATG-3′ (forward), 5′-CTGGTTGATTTTGCGGTATGG-3′ (Reverse) and GAPDH, 5′-GGGGTGAGGCCGGTGCTGAGTAT-3′ (forward), 5′-CATTGGGGTAGGAACACGGAAGG-3′ (reverse). Real- time PCR reactions were performed in the Stratagene Mx3000P QPCR System (Agilent Technologies, Lexington, MA) using SYBR green PCR Master Mix (Froggabio Inc, ON, Canada) and 200 nM of forward and reverse primers. The PCR reaction condition was 95 °C for 10 min, 95 °C for 30 sec, 58 °C for 45 sec, and 72 °C for 30 sec (40 cycles).

### Flow cytometry

Phenotypic analysis characterization of DCs or T cells was performed on a FACS Calibur flow cytometer (Becton Dickinson, San Jose, CA). All antibodies were purchased from eBioscience, San Diego, CA. All flow cytometric analysis was performed using appropriate isotype controls.

T cell and DC subsets were analyzed by means of two- or three-color staining with various combinations of mAbs. DCs were stained with FITC- or PE-CD86, FITC-CD11C, PE- PD-L1 and PE-Cy5-CD40 monoclonal antibodies. For T cells, PE-Cy5-CD4, PE-CD25 and FITC-FoxP3, Percp-efluor710-PD-1 and PE-TIM 3 conjugated anti-mouse monoclonal antibodies were used for staining. Foxp3 expression was assessed by intracellular staining, using a cell permeabilization kit (eBioscience).

### Mixed lymphocyte reaction (MLR)

For *in vitro* MLR, T cells (2 × 10^5^/well) from naïve BALB/c mice were cocultured with DCs cultured and transfected from C57BL/6 mice in different ratios of DC: T cells. For *in vivo* MLR, splenic DCs isolated from tolerant or rejecting recipients (BALB/c) using CD11c MACS beads (MiltenyiBiotec) and cocultured with T cells (2 × 10^5^/well) from C57BL/6 mice in 200 μl of complete RPMI 1640 medium (Life Technologies). Cells were cultured at 37 °C in a humidified atmosphere of 5% CO_2_ for 3 days, and pulsed with 1 μCi of [^3^H] thymidine (PerkinElmer, Woodbridge, ON) for the last 18 h of culture. Cells were harvested and the incorporated radioactivity was quantified using a Wallac Betaplate liquid scintillation counter. Results were expressed as mean ± SEM cpm of triplicate cultures.

### Statistical analysis

In this study, data were reported as the mean ± SEM. Allograft survival among experimental groups was compared using the log-rank test. Quantitative real-time PCR data were analyzed using one-way ANOVA or student’s t-test. Differences with *P* values less than 0.05 were considered significant.

## Additional Information

**How to cite this article**: Wang, H. *et al*. Prevention of allograft rejection in heart transplantation through concurrent gene silencing of TLR and Kinase signaling pathways. *Sci. Rep.* **6**, 33869; doi: 10.1038/srep33869 (2016).
